# Poly(vinylbenzyl chloride-*co*-divinyl benzene) polyHIPE monolith-supported *o*-hydroxynaphthaldehyde propylenediamine Schiff base ligand complex of copper(ii) ions as a catalyst for the epoxidation of cyclohexene†

**DOI:** 10.1039/c9ra05811g

**Published:** 2019-09-30

**Authors:** Keerti Moghe, A. K. Sutar, I. K. Kang, K. C. Gupta

**Affiliations:** Polymer Research Laboratory, Department of Chemistry, Indian Institute of Technology Roorkee Roorkee 247 667 India kcgptfcy@iitr.ac.in keertik@hormail.com +91 1332 273560 +91 132 285325; Department of Polymer Science and Engineering, Kyungpook National University 80, Daehak-ro, Buk-gu Daegu 702-701 South Korea ikkang@knu.ac.kr +82 53 950 6623 +82 53 950 5629

## Abstract

Poly(vinylbenzyl chloride-*co*-divinyl benzene)-based polyHIPE monoliths of different porosities were prepared using high-internal-phase emulsions (HIPEs) containing a fixed amount of vinylbenzyl chloride (VBC, 6.0 g, 0.0393 mol) and divinyl benzene (DVB 4.0 g, 0.0308 mol) as the oil phase and different volume ratios of aqueous calcium chloride as the internal phase. Span-80 (2.0 g (4.67 mmol))-stabilized HIPEs were polymerized at 60 °C using potassium persulfate (0.4 g, 1.48 mmol) as the initiator. Upon varying the volume ratio of aqueous calcium chloride from 80 to 90%, the prepared polyHIPE monoliths have shown significant variations in their surface morphology, specific surface area (SA), and pore volumes (*V*_p_) as confirmed by scanning electron microscopy (SEM) and a gas adsorption (BET) method. The prepared polyHIPE monoliths were anchored with *o*-hydroxynaphthaldehyde propylenediamine Schiff base ligand (HNPn) and then loaded with copper(ii) ions (HNPn–Cu) to act as a catalyst. The structural information of unsupported HNPn–Cu complexes was obtained by recording its FT-IR and UV-visible spectra. The amount of copper(ii) ions loaded onto HNPn ligand-anchored polyHIPE monoliths was determined by atomic absorption spectroscopic analysis. In comparison to unsupported HNPn–Cu catalyst, the polyHIPE monolith-supported HNPn–Cu catalyst has shown high catalytic activity (66.8%), product selectivity for epoxycyclohexane (ECH) (94.8%), high turn over number (0.028 mol mol^−1^ h^−1^) and low energy of activation (22.4 kJ mol^−1^) in the epoxidation of cyclohexene in the presence of hydrogen peroxide (H_2_O_2_) as an oxidant at 40 °C. The polyHIPE-supported HNPn–Cu catalyst also shows high reuse applications. Studies show that there is sufficient scope to develop polyHIPE monoliths with various properties for specific applications.

## Abstract 2

High internal phase emulsion of vinyl benzene and divinyl benzene in aqueous calcium chloride produced porous monoliths,which improved the activity of supported HNPn-Cu catalyst in selective oxidation of cyclohexene in presence hydrogen peroxide.

## Graphical abstract

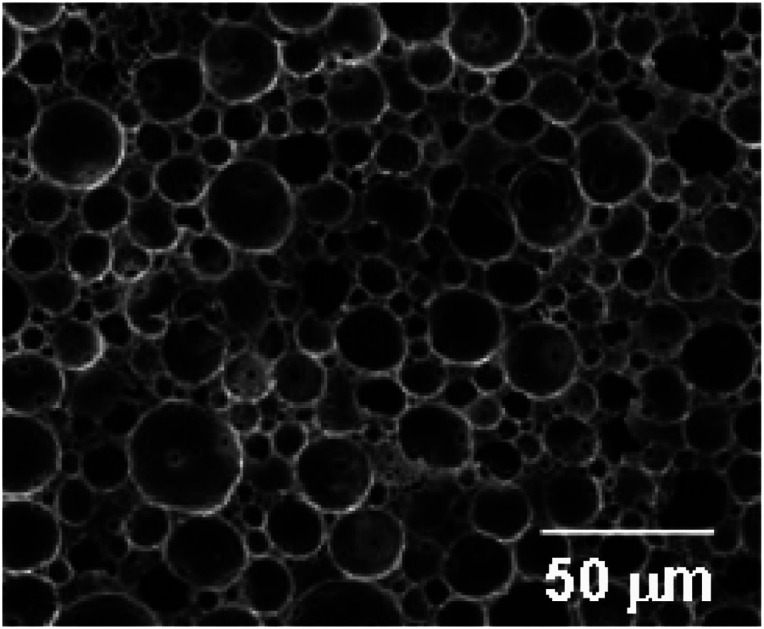

## 1 Introduction

In recent years, the development in synthetic chemistry has taken place at an alarming rate using various types of supported catalysts.^1–10^ The majority of solid-phase syntheses were carried out using polymer gel-bead supports.^2–4,11^ However, in comparison to polymer gel-bead supports, the porous solid supports are found to be better in facilitating the diffusion of reactants to the interior sites of the supports without using swelling media.^5,6^ However, to improve the activity of catalysts on polymer gel-bead, spacers such as polyethylene glycol are used, but cleaving and poor loading of catalyst remained major drawbacks.^3,12–14^ Some efforts were made to increase the loading capacity of supports by controlling their swelling properties and improving the stability of supported catalysts.^4,15^ Property modification in polymer gel-bead supports was found to be more useful in continuous flow reactors than batch reactors.^14,16–19^ However, polymer gel-beads suffered from a channeling effect, which made interior active sites to remain unutilized in flow-through processes.^20^ Considering these drawbacks of polymer gel-bead supports, the polyHIPE monoliths without interstitial space^5,6^ are found to be more useful and ideal for continuous flow reactors.^19,21,22^ To develop porous supports, various techniques such as gas foaming,^23^ porogen leaching,^24^ and techniques of additive manufacturing^25^ are widely used. Nevertheless, these methods remained ineffective in adding inter pore connectivity in porous supports^26^ and were not able to develop well-resolved surface pore morphology.^25^ Thus, highly inter-connected porous polymer monoliths are developed *via* the polymerization of surfactant-stabilized^27–32^ high-internal-phase emulsions (HIPEs) with a volume ratio of dispersed phase of >74%.^33,34^ The polymerization of HIPEs led to the formation of open-cell-structured macro-porous polymer monoliths with sufficiently high surface area and low density.^31,35,36^ These emulsion-templated, interconnected, and open-cell-structured highly porous monoliths^29^ are found to be useful in various applications such as supports for catalysts,^6,37–41^ recovery of residual oils,^42^ media for the storage of gases,^43^ or as an ion exchanger for the separation of metal ions.^21,44^ The nanoparticles supported on polyHIPE monoliths are more efficient^5,45^ due to better flow and mass transport properties of monoliths,^6,46–51^ which is well suited for catalysis.^52^ The surface-functionalized porous polyHIPE monoliths are also found to be useful in solid-phase syntheses and as a reagent scavenger.^53–55^ Though complexes of metal ions on inorganic supports are used as catalysts in various reactions,^56–58^ polymer supports provide a better microenvironment to both reactants and catalysts.^14,37^ Studies have indicated that supported Schiff base complexes of transition metal ions were more catalytic in the epoxidation of olefins^59,60^ than the unsupported ones.^61,62^ To utilize the properties of polyHIPE monoliths, some studies were conducted using physically^63^ or chemically bound catalysts on porous polyHIPE monoliths.^37,63–66^ However, chemically bound catalysts were found to be more efficient and useful as they are free from leaching problems and can be recovered from the reaction mixture. The epoxidation of olefins is a useful process as it can produce intermediates that find potential applications in the synthesis of fine chemicals, and in the production of useful polymers and pharmaceuticals. Though the epoxidation of olefins is possible with organic peroxides or hypochlorites, H_2_O_2_ reduces the formation of unwanted low-valued hazardous waste products. Thus, H_2_O_2_ alone or in combination with molecular oxygen/air is found to be more useful in the epoxidation of alkenes without any environmental problems. The epoxidation of cyclohexene using polymer-supported catalysts has been reported previously,^67–69^ but the epoxidation of cyclohexene using porous polyHIPE monolith-supported Schiff base complex of copper(ii) ions in the presence of hydrogen peroxide as an oxidant has not been reported yet. Thus, in these studies, attempts were made to develop porous polyHIPE monolith-supported copper(ii) ion complex of *o*-hydroxynaphthaldehyde propylenediamine Schiff base ligand (HNPn) as a catalyst for the epoxidation of cyclohexene in the presence of H_2_O_2_ as an oxidant.^37,63^ The complexation of copper(ii) ions for the epoxidation of cyclohexene was carried out using HNPn ligand-functionalized polyHIPE monoliths of different porosities.^70,71^ The pore size and porosity of the prepared polyHIPE monoliths were controlled with different volume ratios of aqueous calcium chloride in high-internal-phase emulsions (HIPEs). The polymerization of HIPEs at 60 °C in the presence of potassium persulfate (K_2_S_2_O_8_) has produced interconnected porous polyHIPE monoliths with uniform morphology as determined by scanning electron microscopy (SEM). To improve the properties of polyHIPE monoliths, hyper cross-linking in polyHIPE monoliths was carried out using dimethoxymethane as the external cross-linker.^72,73^

## 2 Experimental

Vinylbenzyl chloride (VBC), divinyl benzene (DVB, 80%), potassium persulfate (K_2_S_2_O_8_), calcium chloride dihydrate (CaCl_2_·2H_2_O), methanol, and Span-80 (sorbitan monooleate) (HLB = 4.3) were purchased from Sigma Aldrich Chemical Company, USA. All other chemicals were used as received and deionized H_2_O (18.2 mU cm) was obtained using a Millipore Milli-Q Plus water system. The oxidation products of cyclohexene were determined using chlorobenzene as an internal standard and analyzing the reaction mixture using a gas chromatograph equipped with a capillary column (SE-30, 30 m × 0.25 mm × 0.25 μm) and a FID detector.

### 2.1 Preparation of polyHIPE monolith-supported HNPn ligand complex of copper(ii) ions

To prepare polyHIPE-supported HNPn Schiff base ligand complex of copper(ii) ions, firstly, *o*-hydroxynapthaldehyde propylenediamine Schiff base ligand (HNPn) was prepared by taking the alcoholic solution of *o*-hydroxynapthaldehyde (3.44 g, 20 mmol) and 1,2′ propylenediamine (0.741 g, 10 mmol) in a Soxhlet apparatus. The solution was refluxed for about 45 min at 60 °C and then the resultant HNPn ligand was separated after cooling the extract. The separated HNPn ligand (Scheme S1a†) was recrystallized in ethanol, and the resultant yield was 83%. To anchor the prepared HNPn ligand on polyHIPE monoliths, the HNPn ligand was aminofunctionalized by a nitrosation process, which was carried out by reacting 20 mmol (7.65 g, *M*_w_ 382.642 mol^−1^) of HNPn ligand with 20 mmol of sodium nitrite (1.06 g) in 100 mL (1 N) hydrochloric acid at a low temperature (Scheme S1†). After 30 minutes, the prepared *N*,*N*′-bis(4-nitroso-*o*-hydroxy naphthalene)propylenediamine (Scheme S1b†) was filtered and washed with hot and cold water to remove the impurities. The introduced nitroso group in HNPn ligand was reduced by taking 20 mmol (8.812 g, *M*_w_ 440.642 g mol^−1^) of nitrosated HNPn ligand in 50 mL (1.0 N) hydrochloric acid in the presence of metallic iron as a catalyst, which on reduction produced *N*,*N*′-bis(4-amino-*o*-hydroxynaphthalene)propylenediamine Schiff base ligand (AHNPn) as shown in Scheme S1c.† After the preparation of AHNPn, polyHIPE monoliths of different porosities were prepared by mixing 6.0 g VBC (0.0393 mol) and 4.0 g DVB (0.0308 mol) as the oil phase with different volume ratios (80–90%) of aqueous calcium chloride (1.0 g, 6.80 mmol) containing a fixed amount of Span-80 (2.0 g, 4.67 mmol) and potassium persulfate (0.4 g, 1.48 mmol). The resultant mixture after stirring at 300 rpm for about 48 h was polymerized at 60 °C for 1 h in PTEF moulds. PolyHIPE monoliths prepared in the presence of 90, 85 and 80 mL of aqueous calcium chloride were marked as polyHIPE-90, polyHIPE-85, and polyHIPE-80, respectively. To prepare polyHIPE monolith-supported HNPn ligand, 5.0 g (∼2 mm in size) of purified polyHIPE monoliths were refluxed in a Soxhlet apparatus containing a fixed amount of AHNPn (8.17 g, 20 mmol) dissolved in 2-propanol (Scheme S2†). Finally, for the loading of copper(ii) ions, 5.0 g HNPn ligand-functionalized polyHIPE monoliths (polyHIPE-L) were added to an aqueous solution of CuCl_2_ (3.41 g, 20 mmol). PolyHIPE monoliths prepared with different volume ratios of aqueous calcium chloride were functionalized with HNPn ligand (polyHIPE-L) using the same procedure and loaded with copper(ii) ions. To study the effect of meso- and microporosity of polyHIPE monoliths, the HNPn-supported polyHIPE-90 monoliths (polyHIPE-90-L) were hyper cross-linked with dimethoxymethane in the presence of ferric chloride (2.5 g) as a catalyst with 1,2-dichloroethane (50 mL)-swollen polyHIPE-90-L monoliths for 12 h. The HNPn ligand-anchored hyper-cross-linked polyHIPE monoliths (hp-polyHIPE-90-L) were subsequently loaded with copper(ii) ions (hp-polyHIPE-90C).

### 2.2 Characterization of polyHIPE monoliths

#### SEM analysis of polyHIPE monoliths

PolyHIPE monoliths were characterized for the surface morphology and pore size variation by recording their scanning electron micrographs (JEOL JSM 5610 LV). To record scanning electron micrographs, polyHIPE monoliths were attached securely on Al stubs using a double-sided carbon adhesive tape and sputtered with a 10 nm layer of gold.

#### Surface area and pore volume of polyHIPE monoliths

The surface area of polyHIPE monoliths was determined by studying the adsorption of N_2_ gas and using the Brunauer–Emmett–Teller (BET) model. The adsorbed volume of N_2_ at a relative pressure (*P*/*P*_0_) of 0.97 was used to calculate the total pore volume of the prepared polyHIPE monoliths. The porosity (*ε*) of polyHIPE monoliths was determined using the following equation (eqn (1)):1
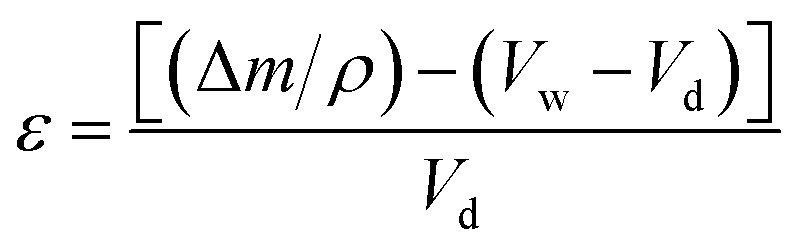
where Δ*m* is the weight of the wetting liquid in monoliths, *ρ* is the density of water used as wetting liquid, and Δ*V* is the volume difference between wet (*V*_w_) and dried (*V*_d_) polyHIPE monoliths.

### 2.3 Characterization of HNPn ligand complex of copper(ii) ions

FT-IR and UV-visible spectra of unsupported HNPn ligand and its copper(ii) ion complex (HNPn–Cu) were recorded using a PerkinElmer 1600 FT-IR Spectrophotometer and a Shimadzu 1601 PC UV-visible Spectrophotometer. Elemental analysis of polyHIPE-L monoliths was carried out using a Haraeus Carlo Ebra 1108 Elemental Analyzer to calculate the loaded amount of HNPn ligand on polyHIPE monoliths. The loaded amount of copper(ii) ions on polyHIPE-L monoliths was estimated by determining the remaining amount of copper(ii) ions in the loading solution using an atomic absorption spectrometer (PerkinElmer 3100) at the *λ*_max_ of copper(ii) ions. The molecular weights of HNPn ligand and its complex with copper(ii) ions were determined using a Vapor Pressure Osmometer (Knauer K-700, Germany) using dimethyl formamide as the solvent and benzyl as the standard. The magnetic moment (*μ*) of HNPn–Cu complex was measured using a vibrating sample magnetometer (VSM-155).

### 2.4 Catalytic activity of polyHIPE monolith-supported HNPn–Cu(ii) ions complex in the epoxidation of cyclohexene

To evaluate the catalytic activity of polyHIPE monolith-supported HNPn–Cu(ii) ions complex as a catalyst in the epoxidation of cyclohexene, 1.g of polyHIPE-supported HNPn–Cu catalyst was placed in a two-necked round-bottom flask containing 2.0 mL acetonitrile, and an equimolar amount (0.05 M) of cyclohexene and hydrogen peroxide (30 wt%) was added. The percent conversion of cyclohexene and product selectivity for epoxycyclohexane (ECH) was evaluated at 40 °C by analyzing the reaction mixture at different time intervals using a gas chromatograph. The epoxidation of cyclohexene was studied using polyHIPE monoliths of different porosities and using different concentrations of cyclohexene, an oxidant (H_2_O_2_), and polyHIPE monolith-supported HNPn–Cu catalyst. The epoxidation of cyclohexene was also studied by varying the reaction temperature from 30 to 50 °C and in the presence of unsupported HNPn–Cu complex as the catalyst. To determine the reuse applications of polyHIPE monolith-supported HNPn–Cu catalyst, the conversion of cyclohexene was also determined using recycled unsupported and polyHIPE monolith-supported HNPn–Cu catalyst.

## 3 Results and discussions

The porous polyHIPE monoliths obtained from high-internal-phase emulsions (HIPEs) are able to overcome the channel-flow problem usually encountered with polymer gel-beads in continuous flow reactors.^74^ Since Schiff base ligand complexes of metal ions are able to catalyze reactions at low temperatures, the polyHIPE monolith-supported Schiff base ligand complex of copper(ii) ions was prepared and used to catalyze the epoxidation of cyclohexene, which is not yet reported in the literature. Various properties of polyHIPE monoliths such as porosity, pore volume, and high surface area^53,73^ were found to be useful in controlling the percent conversion of cyclohexene and the product selectivity of epoxycyclohexane on using polyHIPE monolith-supported HNPn–Cu catalyst. PolyHIPE monoliths have an increasing effect on the activity of supported HNPn–Cu catalyst and increased reuse applications.^75,76^

### 3.1 Poly(vinylbenzyl chloride-*co*-divinyl benzene)polyHIPE monoliths

**Fig. 1:**
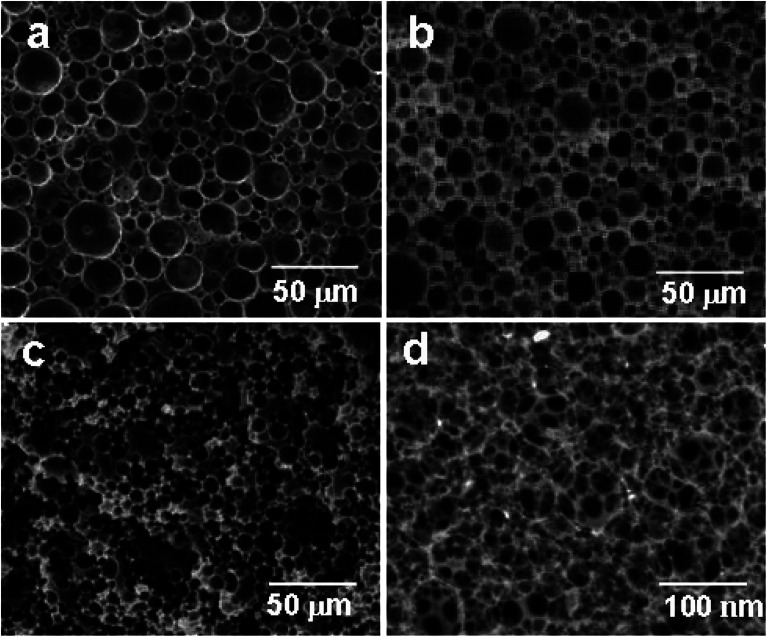
SEM micrographs of polyHIPE monoliths prepared with different volume ratios of aqueous calcium chloride (a) polyHIPE-80, (b) polyHIPE-85, (c) polyHIPE-90; and of hyper cross-linked monoliths (d) hp-polyHIPE-90.

**Fig. 2:**
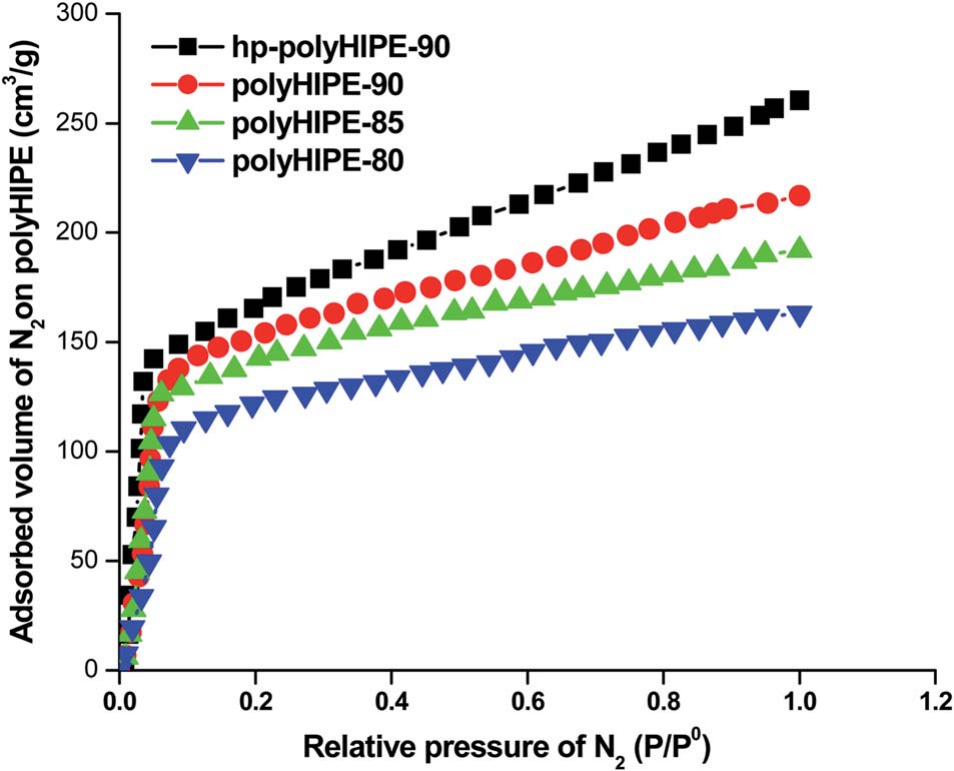
Adsorption isotherms of polyHIPE monoliths prepared with different volume ratios of aqueous calcium chloride.

The high-internal-phase emulsion (HIPE) with a 90% volume ratio of aqueous calcium chloride has produced polyHIPE monoliths (polyHIPE-90) with a porosity of 82.4% along with the pore opening (8–12 μm) at their surfaces (Fig. 1c). In comparison to other polyHIPEs, the polyHIPE-90 monoliths had reduced pore size and high surface area (583.5 m^2^ g^−1^) as determined by the BET method (Table 1). The application of Span-80 (2.0 g, 4.67 mmol) reduced the coalescence of aqueous droplets in the oil phase and helped in the production of stabilized HIPEs.^77^ On decreasing the volume ratio of aqueous calcium chloride in HIPEs to 85% or 80%, a significant variation in the pore size and surface area in the resultant polyHIPE monoliths was clearly observed from their SEM micrographs (Fig. 1a and b) and also supported by their adsorption isotherms (Fig. 2).

**Table 1:** Surface area, pore volume, and average pore size in polyHIPE monoliths

Type of monolith	SA_BET_/m^2^ g^−1^	*V*_p_/cm^3^ g^−1^	Pore size/μm	Porosity (*ε*)/%
hp-polyHIPE-90	619.8	260.5	0.001–0.05	82.4
PolyHIPE-90	583.5	216.4	8–12	76.9
PolyHIPE-85	534.3	190.3	10–15	70.6
PolyHIPE-80	446.3	163.5	15–25	60.2

The pore size in polyHIPE-85 monoliths was varied from 10 to 15 μm (Fig. 1b), whereas polyHIPE-80 monoliths have shown pore size variation from 15 to 25 μm (Fig. 1a). On varying the volume ratio of aqueous phase in HIPEs, a significant variation in the surface area (SA), pore volume (*V*_p_), and porosity (*ε*) was observed (Table 1, Fig. 2). All polyHIPEs were found to be macroporous in nature as their average pore size was larger than 0.05 μm (>50 nm), which helped in a convective flow of reactants and products during the epoxidation of cyclohexene in the presence of hydrogen peroxide as an oxidant.

On polymerization of HIPEs at 60 °C having the volume ratio of aqueous phase from 90 to 80%, the composition of polyHIPE monoliths was found to be almost constant as indicated by the fixed amount of chlorine (∼15.3 ± 3%) in the prepared polyHIPE monoliths. This suggested that during the polymerization of HIPEs, the activity of vinylbenzyl chloride and divinyl benzene monomers did not vary in reaction mixture and both were consumed in a weight ratio of 6 : 4 to produce polyHIPE monoliths with a fixed composition and uniform surface morphology (Fig. 1a–c).

This has also suggested that hydrophilic and lipophilic balance (HLB) of Span-80 was sufficient to stabilize the aqueous-phase droplets in HIPEs prepared by varying the volume ratio of aqueous phase from 80 to 90%.

The hyper cross-linked polyHIPE-90 monoliths (hp-polyHIPE-90) were found to be meso- and microporous (Fig. 1d) due to additional cross-linking reactions between added dimethoxymethane cross linkers and unused pendant chloromethyl groups of poly(vinylbenzyl chloride-*co*-divinyl benzene) copolymers in HNPn ligand-anchored polyHIPE-90 monoliths. However, the pore opening at the surface of hp-polyHIPE-90 monoliths was not sharp (Fig. 1d) as was shown by other polyHIPE monoliths (Fig. 1a–c), which was likely due to high cross-linking provided by adding dimethoxymethane. The hyper-cross-linked polyHIPE monoliths (hp-polyHIPE-90) were having high surface area (619.8 m^2^ g^−1^) and porosity (82%) in comparison to other polyHIPE monoliths (Table 1, Fig. 1 and 2). The observed zero percent of chlorine content and 4.62% content of nitrogen in hp-polyHIPE-90 monoliths have suggested that all unused pendant chloromethyl groups of poly(vinylbenzyl chloride-*co*-divinyl benzene) copolymers in HNPn ligand-anchored polyHIPE-90 monoliths were used up by adding a dimethoxymethane cross-linker.

### 3.2 PolyHIPE monolith-supported HNPn ligand complex of copper(ii) ions

**Fig. 3:**
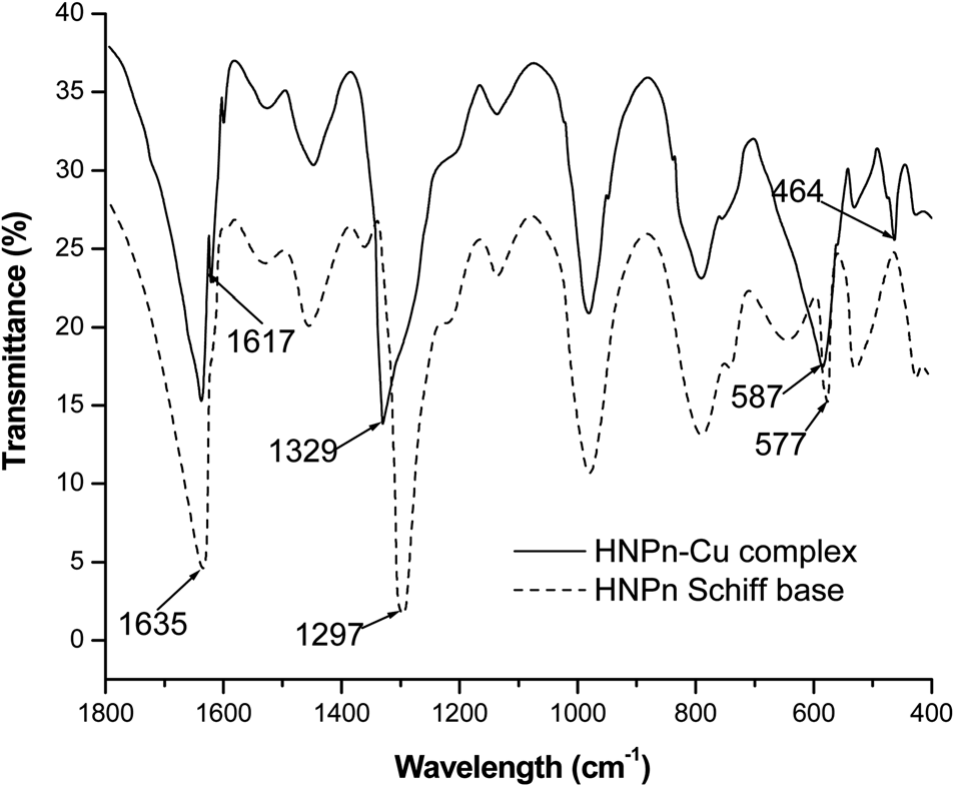
FT-IR spectra of HNPn Schiff base ligand and its copper(ii) ions complex.

**Fig. 4:**
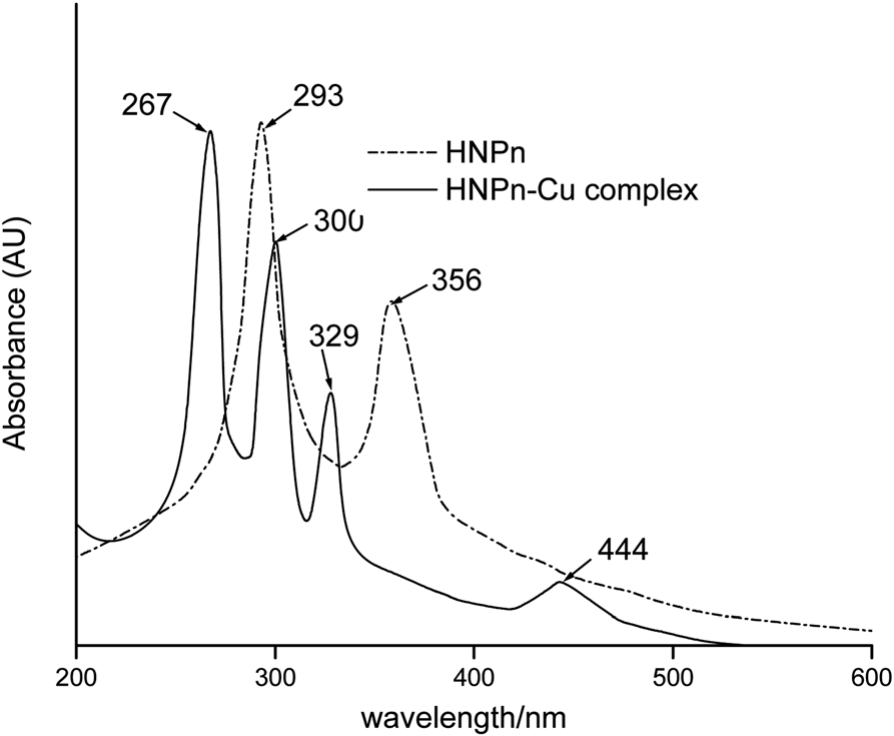
UV-visible spectra of HNPn Schiff base ligand and its copper(ii) ions complex.

The copper(ii) ions loaded polyHIPE monoliths were prepared by reacting HNPn ligand-anchored polyHIPE monoliths with copper(ii) ions. For anchoring the HNPn ligand on polyHIPE monoliths, first of all, HNPn ligand was prepared by reacting *o*-hydroxynapthaldehyde and 1,2′-propylenediamine (Scheme S1a†), which was aminofunctionalized (Scheme S1c†) *via* nitrosation and reduction processes in the presence of FeCl_3_ as a catalyst in an acidic medium (Scheme S1b†). The appearance of absorption bands at 1635 and 1297 cm^−1^ corresponding to –C=N and –C–O groups in the FT-IR spectrum of HNPn ligand (Fig. 3) has supported its formation. Similarly, the appearance of two absorption bands at 293 and 365 nm in the UV-visible spectrum (Fig. 4) corresponding to the π–π* and *n*–π* transitions has confirmed the formation of HNPn Schiff base ligands. The formation of aminofunctionalized HNPn Schiff base ligand (AHNPn) was confirmed by its FT-IR spectrum, which shows absorption bands corresponding to –C=N (1635 cm^−1^) and –C–O (1297 cm^−1^) groups beside a broad band between 3100 and 3150 cm^−1^ corresponding to the primary amine (–NH_2_). The AHNPn ligand was anchored on polyHIPE monoliths (Scheme S2†). PolyHIPE-90 and hyper-cross-linked polyHIPE-90 monoliths (hp-polyHIPE-90) have shown high loading of AHNPn (1.29 mmol) due to high surface area (Table 2) though a fixed amount of AHNPn (20 mmol, 8.17 g) was used for loading with different types of polyHIPE monoliths.

**Table 2:** Loading of HNPn ligand and copper(ii) ions on polyHIPE monoliths^a^

Type of monoliths	HNPn/mmol g^−1^	N_2_/%	Cu(ii) ions/mmol g^−1^	Cl/%
hp-polyHIPE-90	1.29	4.62	1.19	0.02
PolyHIPE-90	1.29	4.89	1.17	3.41
PolyHIPE-85	1.22	4.62	1.04	3.42
PolyHIPE-80	1.15	4.35	0.92	3.44
HNPn-ligand	—	6.92	2.34	—

a Loading amount of HNPn ligand and copper(ii) ions is per gram of polyHIPE monoliths.

The complexation of copper(ii) ions with HNPn ligand was confirmed from the observed shifts in absorption bands from 1635 cm^−1^ to 1617 cm^−1^ (–C=N) and from 1297 cm^−1^ to 1329 cm^−1^ (–C–O) in the FT-IR spectrum of copper(ii) ions complexes of HNPn ligand (Fig. 3). The appearance of two new absorption bands at 587 cm^−1^ and 464 cm^−1^ assigned to –M–O and M–N bonds also supported the complexation of copper(ii) ions with HNPn ligand (Fig. 3). Similarly, the shift in peak positions from 293 nm to 267 nm and from 356 nm to 300 nm corresponding to π → π* and n → π* transitions in the UV-visible spectrum has confirmed the complexation of copper(ii) ions with HNPn ligand (Fig. 4, Scheme S3†). The appearance of two new peaks at 329 nm and 444 nm corresponding to the charge transfer (C–T) and d–d transitions in the UV-visible spectrum has further confirmed the complexation of copper(ii) ions with HNPn ligand (Fig. 4, Scheme S3†). The amount of copper(ii) ions loaded onto unsupported and polyHIPE monolith-supported HNPn ligand was estimated by analyzing the loading solution (20 mmol, 3.41 g) using an atomic absorption spectrometer (*λ*_max_, 324 nm). In comparison to polyHIPE-supported HNPn ligand, unsupported HNPn ligand has shown high complexation of copper(ii) ions (2.34 mmol g^−1^) as shown in Table 2. The magnetic moment (*μ*) of HNPn ligand complex of copper(ii) ions (1.76 BM) has suggested a square planar geometry for HNPn ligand complex of copper(ii) ions (Scheme S3†). The variation in the loading amount of copper(ii) ions on polyHIPE monoliths (Table 2) was due to the variation in the loaded amount of HNPn ligand and variation in the pore size of the prepared polyHIPE monoliths (Table 1). In comparison to polyHIPE-90 monoliths, the hyper cross-linked polyHIPE monoliths (hp-polyHIPE-90) have shown improved complexation of copper(ii) ions (1.19 mmol g^−1^) than polyHIPE-90 monoliths (1.17 mmol g^−1^) due to better opportunities for the binding of copper(ii) ions with HNPn ligand supported on hp-polyHIPE-90 monoliths (Table 2).

### 3.3 Catalytic activity of polyHIPE monolith-supported HNPn ligand complex of copper(ii) ions in the epoxidation of cyclohexene

**Scheme 1:**
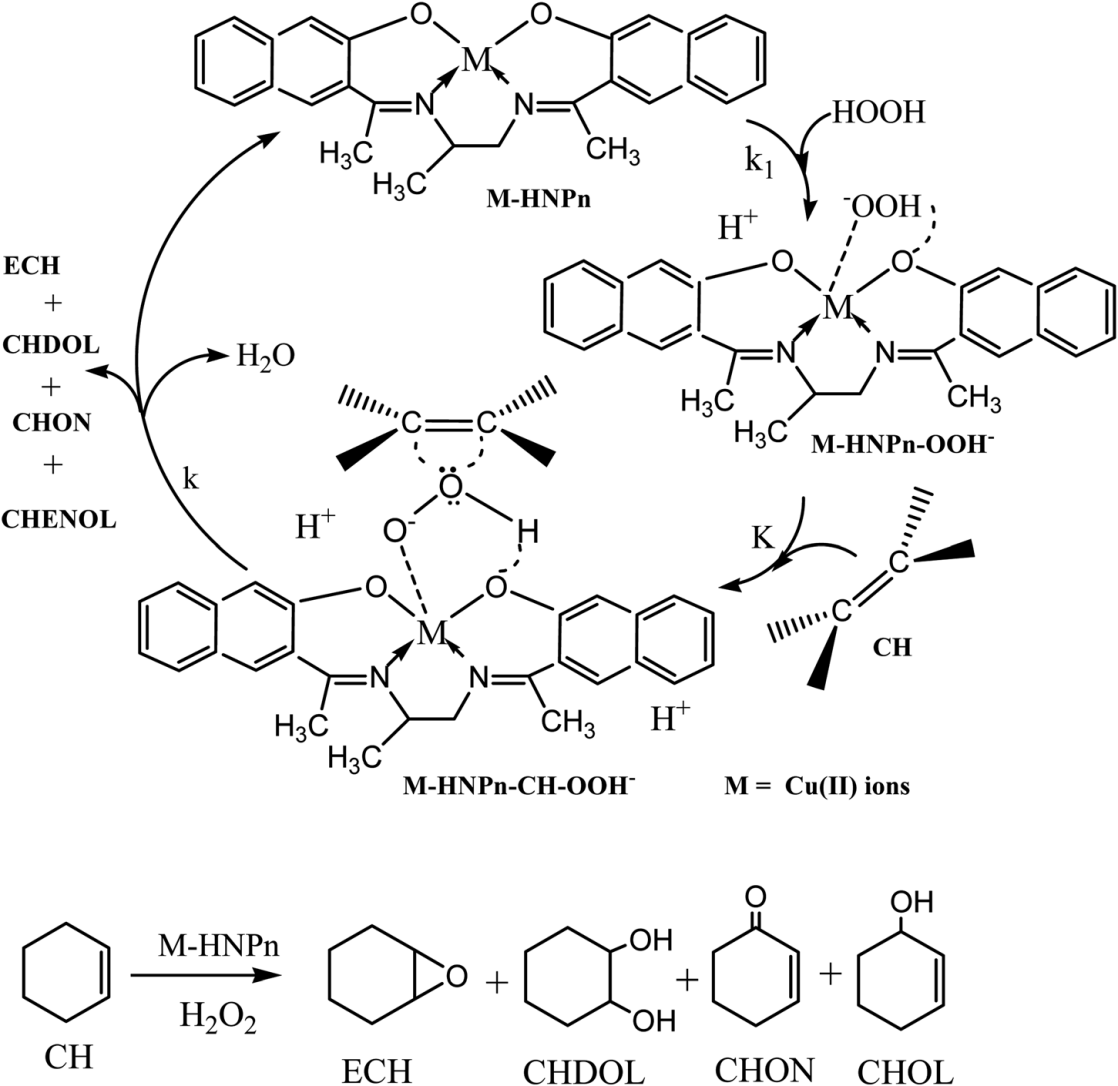
Epoxidation of cyclohexene by HNPn–Cu catalyst in the presence of H_2_O_2_.

To evaluate the effect of polyHIPE monoliths on the activity of anchored HNPn–Cu catalyst, the catalytic activity of unsupported and polyHIPE monolith-supported HNPn–Cu catalyst was evaluated in the epoxidation of cyclohexene in the presence of hydrogen peroxide as an oxidant at 40 °C. The percent conversion of cyclohexene was studied for a fixed time interval (1440 min) and the reaction products were analyzed by gas chromatography. In comparison to unsupported HNPn–Cu catalyst, the polyHIPE monolith-supported HNPn–Cu catalyst was found to be more active and selective for epoxycyclohexane (ECH) than other reaction products such as 2-cyclohexene-1-ol (CHOL), 1,2-cyclohexanediol (CHDOL), and 2-cyclohexene-1-one (CHON). The activity of HNPn–Cu catalyst on polyHIPE monoliths was assumed to be like enzymes and the polyHIPE monolith-supported HNPn–Cu catalyst has shown high activity in the conversion of cyclohexene and the selective production of ECH in comparison to unsupported HNPn–Cu catalyst. Thus, considering the enzymatic action of HNPn–Cu catalyst on polyHIPE monoliths, the formation of active species (M–HNPn–OOH^−^) *via* the interaction of hydrogen peroxide and HNPn–Cu catalyst was presumed to be more prominent than the unsupported HNPn–Cu catalyst (Scheme 1). The resultant active species (M–HNPnOOH^−^) was presumed to involve in the formation of a transient intermediate (M–HNPn–CH–OOH^−^) upon interaction with cyclohexene (CH) through a dynamic equilibrium (*K*) with active species (M–HNPn–OOH^−^). The value of rate constant (*k*_1_) for the formation of active species *via* the interaction of HNPn–Cu catalyst and H_2_O_2_ on polyHIPE monoliths is assumed to be quite higher than that of the unsupported HNPn–Cu catalyst. The high value of *k*_1_ might be responsible to enhance the dynamic equilibrium (*K*) between active species (M–HNPn–OOH^−^) and transient intermediate (M–HNPn–CH–OOH^−^) on polyHIPE monoliths. The high value of equilibrium constant (*K*) on polyHIPE monoliths (Table 3) might be responsible for increasing the rate of oxygen transfer (*k*) to cyclohexene in the selective production of ECH in comparison to the formation of other reaction products such as CHOL, CHDOL, and CHON (Scheme 1).

**Table 3:** Cyclohexene conversion, selectivity for ECH, and kinetic parameters on using polyHIPE monolith-supported and free HNPn–Cu catalyst^a^

Catalysts (CAT)	Conversion (%)	Selectivity (%)	*K*	Δ*S*/J K^−1^ mol^−1^	*E*_a_/kJ mol^−1^
hp-polyHIPE-90C	82.3	97.1	4.65	52.08	12.3
PolyHIPE-90C	66.8	94.8	2.01	76.70	22.4
PolyHIPE-85C	61.8	92.8	1.62	119.66	36.2
PolyHIPE-80C	41.8	88.2	0.72	152.21	48.5
Free HNPn–Cu	54.8	84.2	1.21	246.31	76.6

a [CH] = [CAT] = [H_2_O_2_] = 0.05 M, temp. = 40 °C, time = 1440 min.

**Fig. 5:**
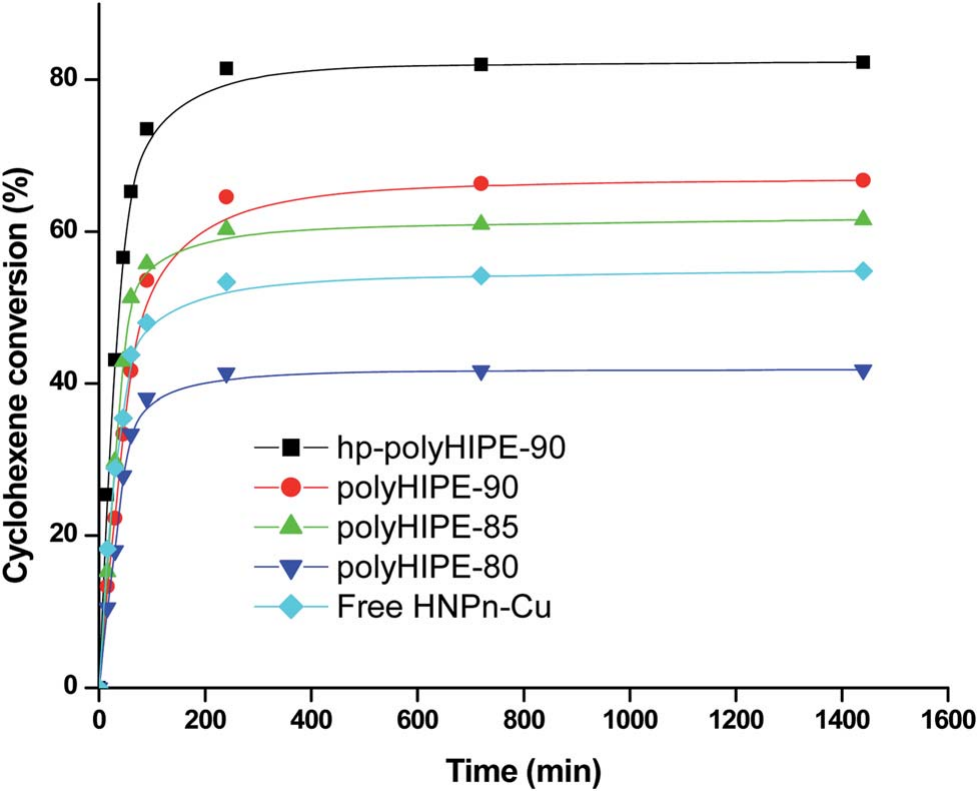
Percent conversion of cyclohexene in the presence of H_2_O_2_ oxidant and on unsupported and polyHIPE monolith-supported HNPn–Cu catalyst.

The results indicated that polyHIPE monoliths were able to provide better microenvironment to the reaction intermediate (M–HNPn–CH–OOH^−^) for its decomposition to form ECH in comparison to the decomposition of an intermediate on unsupported HNPn–Cu catalyst. Thus, the microenvironment provided by polyHIPE monoliths might be a significant factor that increased the activity of HNPn–Cu catalyst on polyHIPE monoliths in the epoxidation of cyclohexene in comparison to unsupported HNPn–Cu catalyst (Fig. 5).

Generally, the oxidation products of cyclohexene are 1,2-cyclohexanediol (CHDOL), 2-cyclohexene-1-one (CHON), 2-cyclohexene-1-ol (CHOL) and epoxycyclohexane (ECH) (Scheme 1), but on using polyHIPE monolith-supported HNPn–Cu catalyst, both percent conversion of cyclohexene and product selectivity for ECH have significantly increased, as the decomposition of the intermediate (M–HNPn–CH–OOH^−^) has followed a route that increased the selective production of ECH for the transfer of oxygen (Scheme 1). In comparison to HNPn–Cu catalyst supported on polyHIPE-85 monoliths, the polyHIPE-90 monolith-supported HNPn–Cu catalyst has shown high activity in the conversion of cyclohexene (66.8%) and its selectivity for ECH (Fig. 5, Table 4). However, the activity of HNPn–Cu catalyst supported on polyHIPE-80 monoliths was found to be lower than unsupported HNPn–Cu catalyst.

**Table 4:** Cyclohexene conversion, selectivity for ECH, and kinetic parameters on using polyHIPE monolith-supported and free HNPn–Cu catalysts^a^

Catalysts (CAT)	Conversion (%)	Selectivity (%)	*R*_p_ × 10^5^/M s^−1^	TON/mol mol^−1^ h^−1^	*E*_a_/kJ mol^−1^
hp-polyHIPE-90C	82.3	97.1	2.38	0.034	12.3
PolyHIPE-90C	66.8	94.8	1.93	0.028	22.4
PolyHIPE-85C	61.8	92.8	1.78	0.026	36.2
PolyHIPE-80C	41.8	88.2	1.21	0.017	48.5
Free HNPn–Cu	54.8	84.2	1.58	0.023	76.6

a [CH] = [CAT] = [H_2_O_2_] = 0.05 M, temp. = 40 °C, time = 1440 min.

The low activity of HNPn–Cu catalyst supported on polyHIPE-80 monoliths was probably due to the low surface area of polyHIPE-80 monoliths (446.6 m^2^ g^−1^) that reduced the loading of copper(ii) ions in comparison to polyHIPE-90 or polyHIPE-85 monoliths. Initially, the percent conversion of cyclohexene was high in the presence of unsupported and polyHIPEs monolith-supported HNPn–Cu catalyst, but after a reaction time of about 100 minutes, the rate of percent conversion of cyclohexenes became slow due to a significant decrease in the remaining amount of cyclohexene and oxidant in the reaction mixture (Fig. 5). However, the overall activity of polyHIPE monolith-supported HNPn–Cu catalyst remained higher than that of unsupported HNPn–Cu catalyst. The HNPn–Cu catalyst on polyHIPE-90 monoliths has shown higher selectivity (94.8%) in the production of ECH in comparison to HNPn–Cu catalyst supported on other types of polyHIPE monoliths (Table 4). The activity of polyHIPE monolith-supported HNPn–Cu catalyst was also analyzed in terms of turn over number (TON), which was found to be higher (0.028 mol mol^−1^ h^−1^) for HNPn–Cu catalyst supported on polyHIPE-90 monoliths in comparison to other types of polyHIPE monoliths and unsupported HNPn–Cu catalyst (Table 4).

Though the activity of HNPn–Cu catalyst supported on polyHIPE monoliths was found to be better than that of unsupported HNPn–Cu catalyst, the activity of HNPn–Cu catalyst supported on hyper-cross-linked polyHIPE monoliths (hp-polyHIPE-90) was found to be significantly high in the percent conversion of cyclohexene, selectivity for ECH, and TON (Fig. 5 and Table 4). This has suggested further that cross-linking in polyHIPE monoliths might be useful in improving the activity of supported catalyst by controlling the porosity and interfacial area of polyHIPE monoliths that help in providing a suitable microenvironment to reactants and the supported catalyst. The observed low values of change in entropy (Δ*S* = 52.08 J K^−1^ mol^−1^) and the energy of activation (*E*_a_ = 12.3 kJ mol^−1^) in the conversion of cyclohexene on using hp-polyHIPE-90 monolith-supported HNPn–Cu catalyst have suggested that cyclohexene was having proper orientation on hp-polyHIPE-90 monoliths in comparison to other types of polyHIPE monoliths and unsupported HNPn–Cu catalyst (Tables 3 and 4). The low catalytic activity of unsupported HNPn–Cu catalyst in the conversion of cyclohexene was due to its tendency to form aggregates in the reaction mixture, which caused a significant decrease in the number of exposed active sites of copper(ii) ions that were essentially needed to catalyze the conversion of cyclohexene. In comparison to unsupported HNPn–Cu catalyst, a large fraction of HNPn–Cu catalyst on polyHIPE monoliths was accessible to catalyze the conversion of cyclohexene and the selective production of ECH.

The percent conversion of cyclohexene and its selectivity for ECH were also studied at 40 °C by varying the molar ratio of cyclohexene from 0.5 to 2 at constant molarity (0.05 M) of hydrogen peroxide and HNPn–Cu catalyst. The results have indicated that the percent conversion of cyclohexene increased linearly on increasing the molar ratio of cyclohexene from 0.5 to 1.0 on using unsupported and polyHIPE monolith-supported HNPn–Cu catalyst (Fig. S1,†Table 5). However, on further increasing the molar ratio of cyclohexene to 2.0, a decreasing trend in the percent conversion of cyclohexene was observed, which might be due to the absence of sufficient amount of oxidant and catalyst in the reaction mixture at high molar ratio (2.0) of cyclohexene. On varying the molar ratio of cyclohexene in the presence of unsupported and polyHIPE monolith-supported HNPn–Cu catalyst, the selectivity for ECH remained almost constant (Table 5). The conversion of cyclohexene and its product selectivity for ECH were also studied at 40 °C on varying the molar ratio of H_2_O_2_ from 0.5 to 2 at a constant molarity (0.05 M) of cyclohexene and the catalyst in the reaction mixture. The trend of the percent conversion of cyclohexenes (Fig. S2†) and its selectivity for ECH (Table 5) during molar ratio variation of H_2_O_2_ in the presence of unsupported and polyHIPE monolith-supported HNPn–Cu catalyst was found to be almost the same as was found during the molar ratio variation of cyclohexene (Fig. S1,†Table 5). In contrast to the effect of molar ratio variation of cyclohexene and hydrogen peroxide, and the molar ratio variation of unsupported and polyHIPE-supported HNPn–Cu catalyst from 0.5 to 1.0, the rate of conversion of cyclohexene at 40 °C has shown an increasing trend along with the increased selectivity for ECH, while keeping the molarity of H_2_O_2_ and cyclohexene (0.05 M) in the reaction mixture constant (Fig. S3,†Table 6). The value of TON at a molar ratio of 0.5 of unsupported and polyHIPE monolith-supported HNPn–Cu catalysts wa found to be higher than that observed at a molar ratio of 1.0 or 2.0, which was probably due to the high rate of conversion of cyclohexene at a molar ratio of 0.5 (Table 6).

**Table 5:** Effect of CH and H_2_O_2_ concentration on the selectivity for ECH (%)^a^

Catalyst (CAT)	% selectivity for ECH at different concentrations of CH			% selectivity for ECH at different concentrations of H_2_O_2_		
	0.025 M	0.050 M	0.10 M	0.025 M	0.050 M	0.10 M
hp-polyHIPE-90C	96.8	97.1	97.0	97.0	97.1	97.1
PolyHIPE-90C	94.7	94.8	94.8	94.6	94.8	94.0
PolyHIPE-85C	92.6	92.8	92.7	92.6	92.8	92.6
PolyHIPE-80C	86.2	88.2	88.0	86.0	88.2	87.8
Free-HNPn–Cu	83.1	84.2	83.8	83.6	84.2	83.8

a [CAT] = 0.05 M, temp. = 40 °C, time = 1440 min. CH_3_CN = 20 mL.

**Table 6:** Effect of catalyst concentration on TON and conversion rate of CH^a^

Catalyst (CAT)	Concentration of HNPn catalyst in reaction mixture					
	0.025 M		0.050 M		0.10 M	
	TON/mol mol^−1^ h^−1^	*R*_p_/10^5^ M s^−1^	TON/mol mol^−1^h^−1^	*R*_p_/10^5^ M s^−1^	TON/mol mol^−1^ h^−1^	*R*_p_/10^5^ M s^−1^
hp-polyHIPE-90C	0.058	2.03	0.034	2.38	0.017	2.30
PolyHIPE-90C	0.051	1.80	0.028	1.93	0.013	1.83
PolyHIPE-85C	0.041	1.44	0.026	1.78	0.012	1.72
PolyHIPE-80C	0.032	1.12	0.017	1.21	0.011	1.45
Free-HNPn–Cu	0.038	1.34	0.023	1.59	0.011	1.55

a [CH] = [H_2_O_2_] = 0.05 M, temp. = 40 °C, time = 1440 min, CH_3_CN = 20 mL.

The decreasing trend in the efficiency of unsupported HNPn–Cu catalyst at high molar ratio was due to its tendency to aggregate in the reaction mixture, whereas a decreasing trend in the efficiency or TON at a higher molar ratio of polyHIPE-supported HNPn–Cu catalyst was due to a fixed porosity or pore volume of polyHIPE monoliths that limited the available amount of cyclohexene for its conversion. The selectivity variation for ECH during the molar ratio variation of unsupported and polyHIPE monolith-supported HNPn–Cu catalyst has clearly indicated that polyHIPE monoliths have played a significant role in controlling the route of decomposition of the proposed intermediate (M–HNPn–CH–OOH^−^) in the selective formation of ECH (Scheme 1). The rate of conversion of cyclohexene was also determined by varying the temperature from 30 to 50 °C at a constant molarity (0.05 M) of cyclohexene, H_2_O_2_ and HNPn–Cu catalyst in CH_3_CN (Table 7). The rate of conversion of cyclohexene has shown an increasing trend on increasing the reaction temperature from 30 to 40 °C, but on increasing the temperature beyond 40 °C, it has shown a decreasing trend. This was due to the decomposition of hydrogen peroxide at high temperature, which reduced the formation of activated species (M–HNPn–OOH^−^) and reaction intermediates (M–HNPn–CH–OOH^−^) in the reaction mixture (Table 7).

**Table 7:** Temperature *versus R*_p_ for CH and Δ*E*_a_ on different polyHIPE monoliths^a^

Catalyst (CAT)	30 °C		40 °C		50 °C		Δ*E*_a_/kJ mol^−1^
	Conv/%	*R*_p_ × 10^7^/M s^−1^	Conv/%	*R*_p_ × 10^5^/M s^−1^	Conv/%	*R*_p_ × 10^5^/M s^−1^	
hp-polyHIPE-90C	69.8	9.1	82.3	2.38	80.2	1.44	12.24
PolyHIPE-90C	59.2	8.1	66.8	1.93	62.6	1.20	22.4
PolyHIPE-85C	45.3	6.1	61.6	1.78	58.2	0.92	36.2
PolyHIPE-80C	36.5	4.9	41.8	1.21	39.4	0.11	48.5
Free HNPn–Cu	40.2	3.4	54.8	1.59	44.6	0.52	76.6

a [CH] = [H_2_O_2_ ] = [CAT] = 0.05 M, time = 1440 min, CH_3_CN = 20 mL.

The selectivity for ECH was increased till 40 °C, but shows a decreasing trend on further increasing the reaction temperature beyond 40 °C (Fig. S4†). This was attributed to a significant retardation in the properties of the transient intermediate (M–HNPn–CH–OOH^−^) upon increasing the reaction temperature beyond 40 °C. The variation in the reaction rate (*k*) on varying the reaction temperature was used to calculate the energy of activation (Δ*E*_a_) for the conversion of cyclohexene, which was found to be low for polyHIPE-supported HNPn–Cu catalyst and also showed dependence on the type of polyHIPE monoliths used for supporting the HNPn–Cu catalyst. The comparison of change in entropy (Δ*S*), equilibrium constant (*K*), and energy of activation (Δ*E*_a_) for the conversion of cyclohexene has provided a concrete proof that polyHIPE monoliths were able to provide a suitable microenvironment to the supported HNPn–Cu catalyst to catalyze the conversion of cyclohexene and the selective production of ECH in comparison to the unsupported HNPn–Cu catalyst (Tables 3 and 7).

### 3.4 Reuse applications of polyHIPE monolith-supported HNPn–Cu catalyst in the epoxidation of cyclohexene

**Fig. 6:**
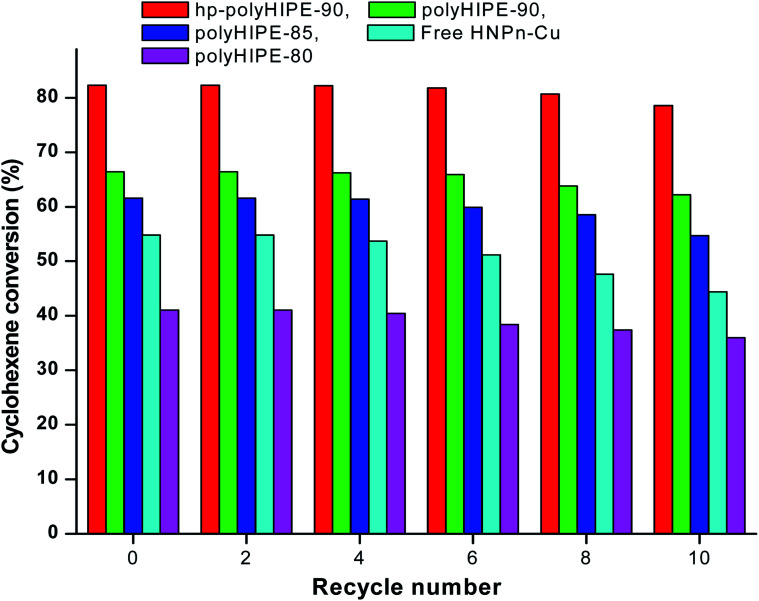
Effect of recycling on the activity of catalyst in the epoxidation of cyclohexene.

To determine the reuse applications of unsupported and polyHIPE monolith-supported^38^ HNPn–Cu catalyst, the activity of the catalyst was evaluated after recycling the HNPn–Cu catalyst. The activity of the HNPn–Cu catalyst on hypercross-linked monoliths (hp-polyHIPE-90) remained almost 99% to its original activity on recycling it for about 8 times, whereas the HNPn–Cu catalyst supported on polyHIPE-90 monoliths was able to show almost the same activity after recycling for six times (Fig. 6).

However, HNPn–Cu catalyst supported on polyHIPE-85 or polyHIPE-80 monoliths were able to show the same activity on recycling it for about four times. The unsupported HNPn–Cu catalyst showed a decreasing trend in its activity after recycling for two times (Fig. 6). The HNPn–Cu catalyst on polyHIPE monoliths retained its structural stability on recycling for about six times, but the structural stability of unsupported HNPn–Cu catalyst seems to be lost after recycling for two times. Thus, polyHIPE monoliths have not only increased the activity of HNPn–Cu catalyst but also increased their stability and reuse applications.^78^

## 4 Conclusions

High-internal-phase emulsions (HIPEs) have been prepared at room temperature by vigorously stirring different volume ratios of aqueous calcium chloride (80–90%) in an organic phase containing a fixed amount of vinylbenzyl chloride (VBC) and divinylbenzene (DVB) in the presence of Span-80. The polymerization of HIPEs with different volume ratios of aqueous calcium chloride in the presence of potassium persulfate as an initiator at 60 °C has produced polyHIPE monoliths with different surface areas, pore volumes, and pore sizes. The polyHIPE monoliths have been functionalized with o-hydroxy napthaldehyde propylenediamine Schiff base ligands (HNPn) and then loaded with copper(ii) ions to act as a catalyst (HNPn–Cu) for the epoxidation of cyclohexene in the presence of hydrogen peroxide as an oxidant at 40 °C. The polyHIPE monoliths prepared using 90% volume ratio of aqueous calcium chloride (polyHIPE-90) were having high surface area, porosity, and capability to load high amounts of copper(ii) ions in comparison to polyHIPE monoliths prepared with 85% (polyHIPE-85) or 80% (polyHIPE-80) volume ratios of aqueous calcium chloride. The polyHIPE monolith-supported HNPn–Cu catalysts were found to be highly active in the conversion of cyclohexene and in the selective formation of epoxycyclohexane (ECH) as confirmed from the obtained values of equilibrium constant (*K*), turn over number (TON), and energy of activation (*E*_a_). The activity of polyHIPE monolith-supported HNPn–Cu catalyst was explained by proposing a scheme similar to the activities of enzymes. The percent conversion of cyclohexene and the selectivity for ECH were found to vary significantly on varying the molar ratio of HNPn–Cu catalyst and reaction temperature, but the selectivity for ECH remained almost constant on varying the molar ratio of cyclohexene and H_2_O_2_ in the reaction mixture. These trends have suggested that the conversion of cyclohexene and the selectivity for ECH was influenced significantly by the microenvironment of polyHIPE monoliths that modified the interactions of the supported HNPn–Cu catalyst with cyclohexene for its conversion and product selectivity of ECH in comparison to the unsupported HNPn–Cu catalyst. The polyHIPE monoliths helped in increasing the stability and reuse application of supported HNPn–Cu catalysts. These studies have clearly indicated that porous polyHIPE monoliths are very useful in modifying the activity of catalyst and have sufficient scope in catalysis and other applications.

## Author contributions

Keerti Moghe and A. K. Sutar were research students at Department of Chemistry, IIT Roorkee and helped partly in collection of experimental data. The kinetics and characterization studies were carried out by Prof. K. C. Gupta during his visit to Department of Polymer Science and Engineering, Kyungpook National University, South Korea. Prof. Inn-Kyu Kang participated in discussions and improving the draft of this manuscript.

## Conflicts of interest

The authors declare no competing financial interest.

## Supplementary Material

RA-009-C9RA05811G-s001
