# Supplementary material for: Poly(vinylbenzyl chloride-co-divinyl benzene) polyHIPE monolith-supported o-hydroxynaphthaldehyde propylenediamine Schiff base ligand complex of copper(ii) ions as a catalyst for the epoxidation of cyclohexene
Source: RSC Adv. 2019 Sep 30;9(53):30823–34. doi: 10.1039/c9ra05811g (PMC9072203; doi:10.1039/c9ra05811g)
Supplement: RA-009-C9RA05811G-s001 [file RA-009-C9RA05811G-s001.pdf]

Electronic Supplementary Material (ESI) for RSC Advances.
This journal is © The Royal Society of Chemistry 2019

Supplementary materials

1-Preparation of HNPn and AHNPn Schiff base ligand

The N, N'-bis (o-hydroxy naphthalene) propylenediamine Schiff base ligand (HNPn) was prepared by reacting o-hydroxynapthaldehyde (3.44 g, 20 mmol) and 1, 2' propylene diamine (0.741 g, 10 mmol) after refluxing for about 45 min at 60 ^{o}C in Soxhlet apparatus (Scheme S1a). The resultant HNPn ligand was amino functionalized by nitrosation process carried out using 20 mmol (7.65 g, Mw 382.642/gmol) of HNPn and 20 mmol of sodium nitrite (1.06 g) in 100 mL of hydrochloric acid (1.0 N) at low temperature. The prepared N, N'-bis (4-nitroso-o-hydroxy naphthalene) propylenediamine (Scheme S1b) was filtered and washed with hot and cold water to remove impurities. The resultant N, N'-bis (4-nitroso-o-hydroxy naphthalene) propylenediamine was reduced by taking 20 mmol (8.812 g) of nitrosated HNPn ligand in 50 mL (1.0 N) hydrochloric acid in presence of metallic iron as catalyst, which produced N, N'-bis (4-amino-o-hydroxynaphthalene) propylenediamine Schiff base ligand (AHNPn) as shown in Scheme S1c.

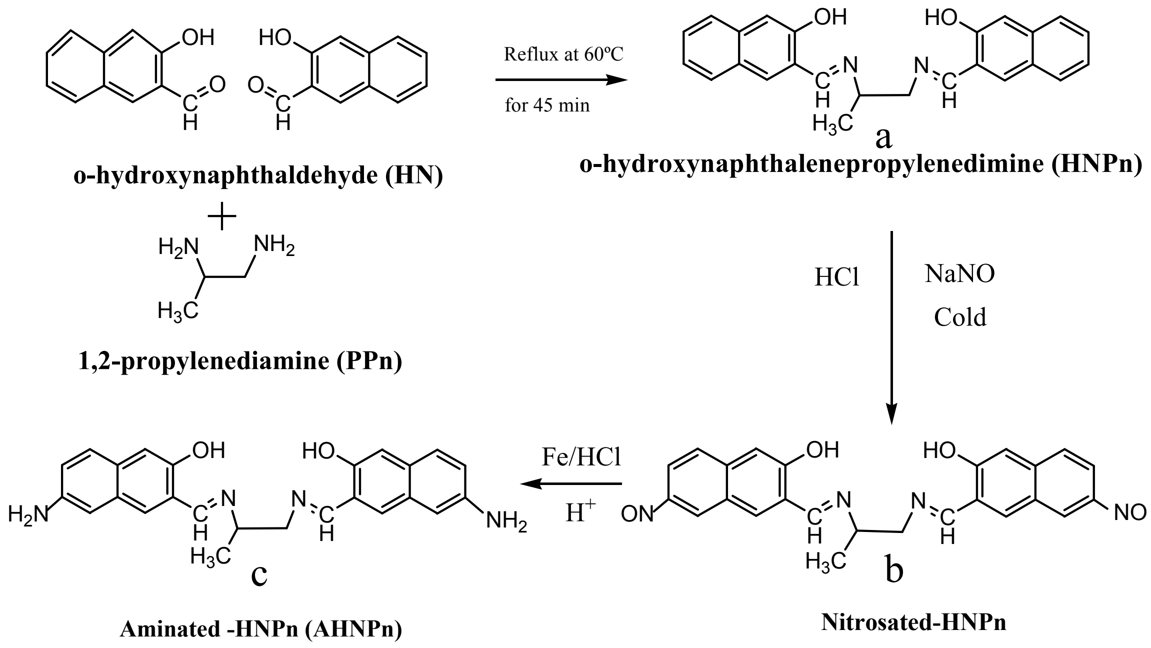

Scheme S1. Synthesis of aminofunctionalized HNPn Schiff base (AHNPn).

2-Preparation of polyHIPE monoliths supported HNPn ligand complex of copper (II) ions

The amino functionalized HNPn ligand (AHNPn) was anchored on polyHIPE monoliths, by taking 5.0 g (~2 mm in size) methanol swollen polyHIPE monoliths in Soxhlet apparatus along with alcoholic solution of AHNPn Schiff base ligand (20 mmol, 8.17g) and mixture was refluxed at 60 ^{0}C (Scheme S2). After refluxing for 6 h, the HNPn ligand functionalized polyHIPE monoliths were separated and purified with propanol and deionized water (Scheme S2).

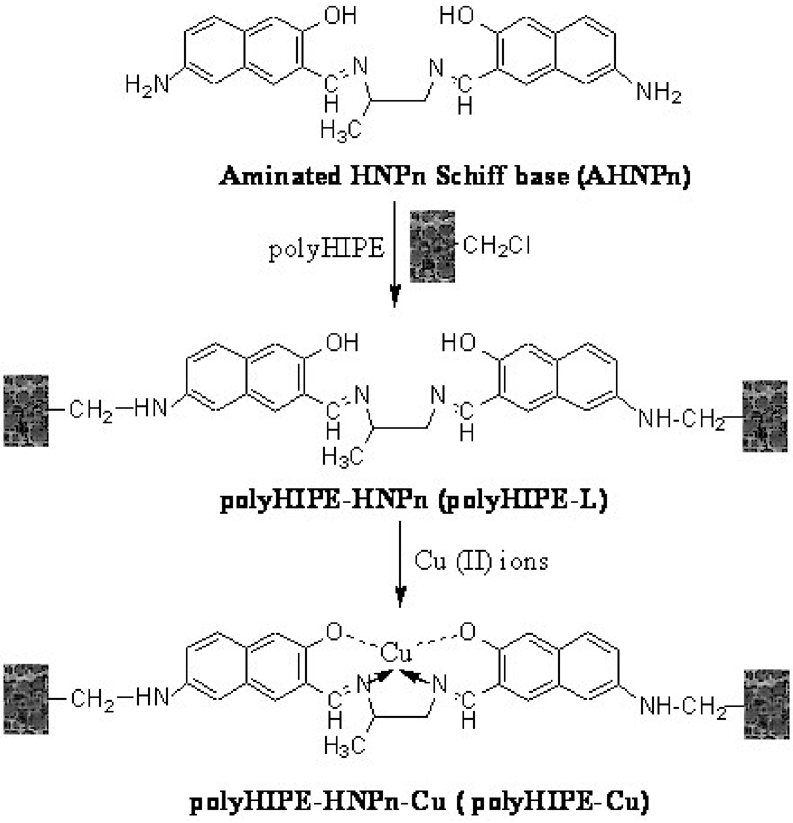

Scheme S2: Anchoring of AHNPn ligand on polyHIPE monoliths and complexation of copper (II) ions.

Finally, 5.0 g of HNPn functionalized polyHIPE monoliths (polyHIPE-L) were allowed to react with aqueous $CuCl_2$ (3.41g, 20 mmol) for loading of copper (II) ions with polyHIPE monoliths supported HNPn ligand (Scheme S2). Similar procedure used for loading of copper (II) ions on polyHIPE monoliths prepared with different volume ratio of aqueous calcium chloride and loading on unsupported HNPn ligand (Scheme S3). The

magnetic moment (μ) has suggested a square planar geometry for complex of copper (II) ions.

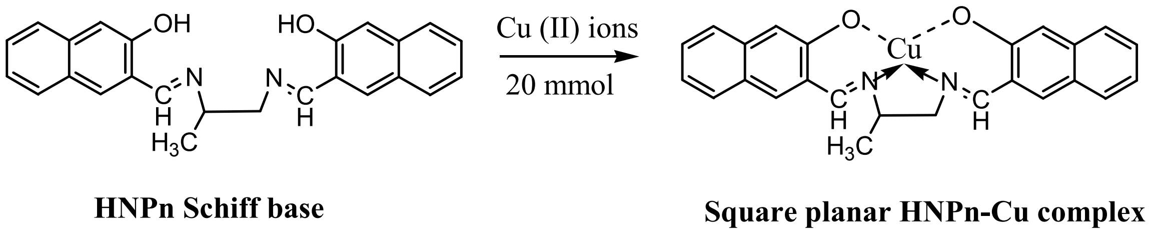

Scheme S3. Complexation of copper (II) ions with unsupported HNPn Schiff base ligand.

3-Epoxidation of cyclohexene using unsupported and polyHIPE monoliths supported HNPn ligand complex of copper (II) ions

Effect of molar ratio of cyclohexene and hydrogen peroxide on conversion of cyclohexene

The percent conversion of cyclohexene was studied by varying the molar ratio of cyclohexene and hydrogen peroxide from 0.5 to 2 at constant molarity of HNPn-Cu catalyst (0.05 M) at 40 °C.

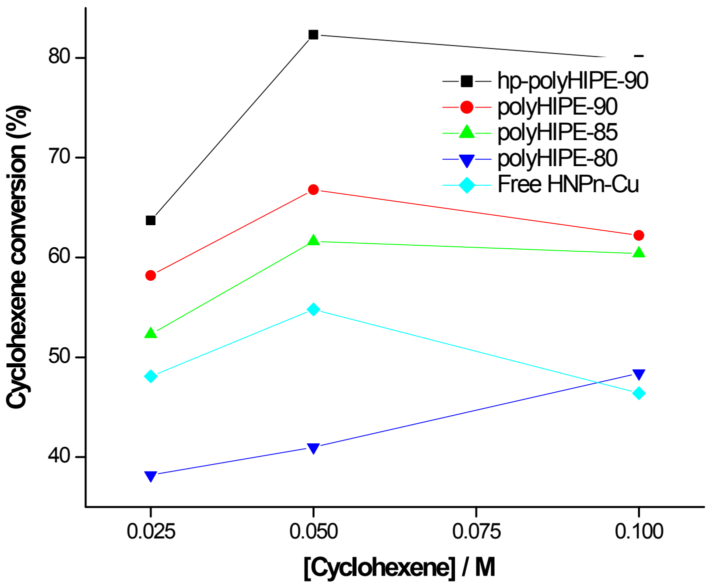

Fig. S1: Effect of molar ratio of cyclohexene on its conversion in presence of unsupported and polyHIPE monoliths supported HNPn-Cu catalyst

The percent conversion of cyclohexene has increased on increasing the molar ratio of cyclohexene (Fig. S1) and hydrogen peroxide from 0.5 to 1.0 (Fig. S2). However, on further increasing the molar ratio of cyclohexene and hydrogen peroxide beyond 1.0, the percent conversion of cyclohexene has shown a decreasing trend. The selectivity for epoxy cyclohexene did not vary on varying the molar ratio of cyclohexene and hydrogen peroxide.

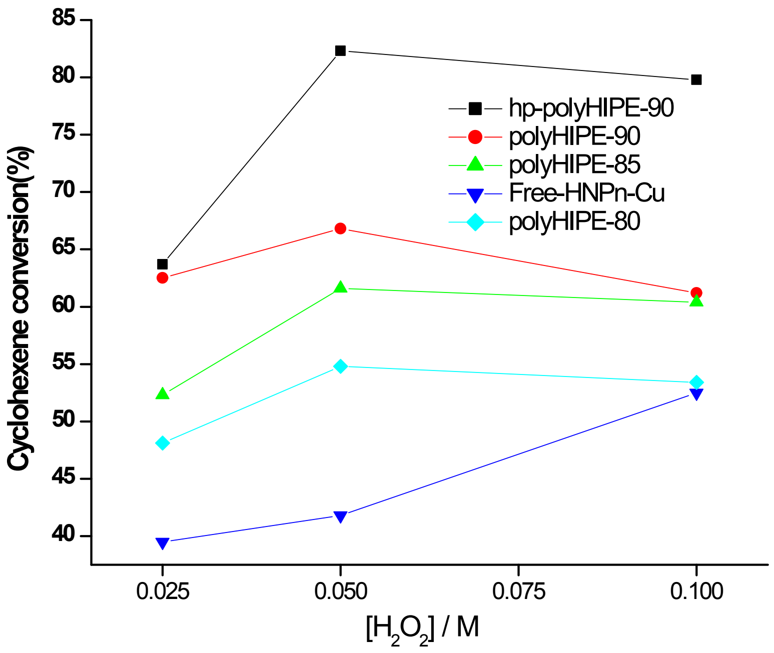

Fig. S2 Effect of molar ratio of H_2O_2 on conversion of cyclohexene in presence of unsupported and polyHIPE monoliths supported HNPn-Cu catalyst.

Effect of molar ratio of catalyst and reaction temperature on selectivity for ECH

The percent conversion of cyclohexene was studied by varying the molar ratio of catalyst from 0.5 to 2.0 at constant molarity of H_2O_2 and cyclohexene (0.05 M) in reaction mixture at 40 ^{0}C. On varying the molar ratio of unsupported and polyHIPE supported HNPn-Cu catalyst from 0.5 to 1.0, the selectivity for epoxycyclohexane (ECH) (Fig. S3) has shown increasing trend. Similar trend for selectivity for ECH (Fig. S4) was observed during temperature variation from 30-50 ^{o}C while keeping constant molarity (0.5M) of cyclohexene, H_2O_2 and HNPn-Cu catalyst in reaction mixture.

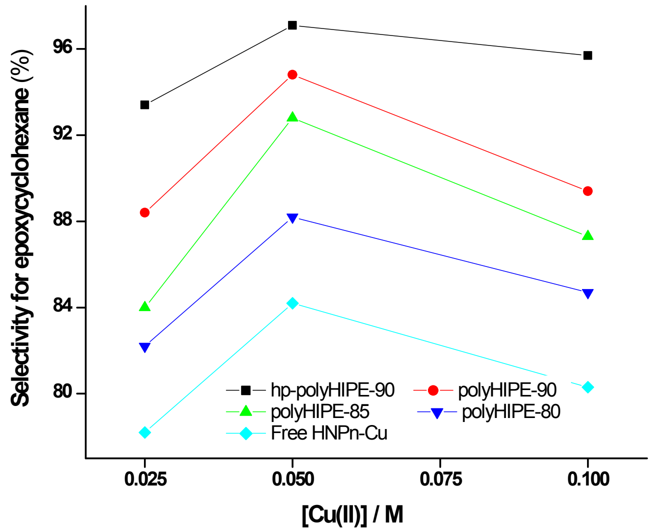

Fig. S3. Effect of molarity of catalyst on selectivity for epoxy cyclohexane in presence of unsupported and polyHIPE monoliths supported HNPn-Cu catalyst.

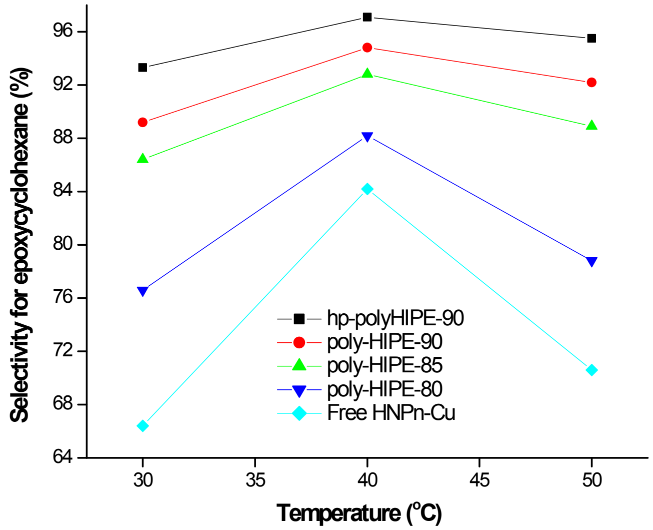

Fig. S4 Effect of reaction temperature on selectivity for epoxy cyclohexane in presence of unsupported and polyHIPE monoliths supported HNPn-Cu catalyst.
